# Automated harvesting and processing of protein crystals through laser photoablation

**DOI:** 10.1107/S2059798316000954

**Published:** 2016-03-24

**Authors:** Ulrich Zander, Guillaume Hoffmann, Irina Cornaciu, Jean-Pierre Marquette, Gergely Papp, Christophe Landret, Gaël Seroul, Jérémy Sinoir, Martin Röwer, Frank Felisaz, Sonia Rodriguez-Puente, Vincent Mariaule, Peter Murphy, Magali Mathieu, Florent Cipriani, José Antonio Márquez

**Affiliations:** a Grenoble Outstation, European Molecular Biology Laboratory; Unit of Virus Host-Cell Interactions (UMI 3265), University Grenoble Alpes–EMBL–CNRS, 71 Avenue des Martyrs, 38042 Grenoble, France; b Structure Design Informatics and Structural Biology, Sanofi, 13 Quai Jules Guesde, 94403 Vitry-sur-Seine, France

**Keywords:** automated crystal mounting, automated cryocooling, crystal soaking, ligand screening

## Abstract

Currently, macromolecular crystallography projects often require the use of highly automated facilities for crystallization and X-ray data collection. However, crystal harvesting and processing largely depend on manual operations. Here, a series of new methods are presented based on the use of a low X-ray-background film as a crystallization support and a photoablation laser that enable the automation of major operations required for the preparation of crystals for X-ray diffraction experiments. In this approach, the controlled removal of the mother liquor before crystal mounting simplifies the cryocooling process, in many cases eliminating the use of cryoprotectant agents, while crystal-soaking experiments are performed through diffusion, precluding the need for repeated sample-recovery and transfer operations. Moreover, the high-precision laser enables new mounting strategies that are not accessible through other methods. This approach bridges an important gap in automation and can contribute to expanding the capabilities of modern macromolecular crystallography facilities.

## Abstract 2

New methods for crystal mounting, soaking and cryocooling contribute to bridging the automation gap between crystallization and X-ray data collection.

## 1. Introduction   

The development of highly focused, automated synchrotron X-ray beamlines and the generalized use of crystallization robots has contributed to facilitating the structural analysis of very challenging targets (Abola *et al.*, 2000 ▸; Banci *et al.*, 2006 ▸; Edwards, 2009 ▸; Rupp *et al.*, 2002 ▸; Cipriani *et al.*, 2006 ▸; Cusack *et al.*, 1998 ▸) and to accelerating the analysis of small molecule–protein interactions by X-ray crystallography, which is commonly applied in drug-design programs (Whittle & Blundell, 1994 ▸; Blundell *et al.*, 2002 ▸). However, the process of preparing crystals for diffraction experiments largely remains a manual and delicate operation that can result in sample loss or degradation of diffraction properties. Crystals are typically harvested by scooping them from the crystallization solution in which they grow with the help of a loop or mesh at the tip of an X-ray data-collection pin and are flash-cooled to cryogenic temperatures in order to reduce radiation damage during data collection (Teng, 1990 ▸; Thorne *et al.*, 2003 ▸; Garman, 1999 ▸). To prevent ice formation, crystals often need to be treated before flash-cooling through a variety of cryoprotection protocols, the most common of which involves the sequential recovery and transfer of the crystals to one or multiple solutions containing antifreezing agents (Garman, 1999 ▸; Garman & Schneider, 1997 ▸). A similar protocol, called soaking, is used to deliver other chemicals to crystals, such as small molecules or phasing agents. This approach is often used in the context of structure-guided drug-design campaigns, in which multiple chemical compounds are screened through crystal soaking, resulting in the need to prepare and analyse hundreds of crystal samples (Blundell *et al.*, 2002 ▸; Davies & Tickle, 2012 ▸). A simplified protocol for sample cryocooling, which consists of the manual removal of the solution surrounding the crystal with a paper wick, has successfully been applied to samples that would otherwise require the addition of cryoprotectants (Pellegrini *et al.*, 2011 ▸). However, crystal mounting remains a critical step that is difficult to apply to small-sized or fragile crystals, and finding optimal protocols for cryocooling and soaking for a particular sample is not always straightforward. Recently, the introduction of fast pixel-array detectors has enabled new experimental approaches and contributed to a notable increase in the sample-processing capacity of modern X-ray beamlines (de Sanctis *et al.*, 2012 ▸; Hülsen *et al.*, 2006 ▸). However, it has also introduced new challenges for the operation of macromolecular crystallography (MX) facilities, with sample-exchange cycles currently taking longer than X-ray measurements. Manual crystal-mounting and processing methods may be insufficient to exploit the full potential of modern MX facilities. A number of approaches have been proposed to automate crystal harvesting and to facilitate sample delivery to X-ray beams, including the use of operator-assisted micromanipulator robots (Viola *et al.*, 2007 ▸, 2011 ▸; Heidari Khajepour *et al.*, 2013 ▸), optical tweezers (Wagner *et al.*, 2013 ▸) and acoustic drop delivery (Yin *et al.*, 2014 ▸), among others (Deller & Rupp, 2014 ▸). Nevertheless, no single approach has yet addressed the multiple manipulations required for the preparation of crystals for diffraction experiments (Deller & Rupp, 2014 ▸).

We have previously shown that the transfer of crystals to X-ray data-collection pins can be automated through the so-called CrystalDirect approach, which relies on the use of a thin, low X-ray-background film as a crystallization support from which crystals are recovered by excising the film with a laser beam and attaching it to an X-ray data-collection pin (Cipriani *et al.*, 2012 ▸; Márquez & Cipriani, 2014 ▸). This approach provides a new level of control over the sample-mounting process that we have exploited here to develop new methods enabling critical crystal manipulations, including crystal cryocooling and crystal soaking.

## 2. Experimental procedures   

### 2.1. Protein production and crystallization   

Crystallization experiments were carried out at the High Throughput Crystallization Laboratory of the EMBL Grenoble Outstation (https://embl.fr/htxlab) using the sitting-drop vapour-diffusion method and CrystalDirect plates (MiTeGen, Ithaca, USA) based on crystallization conditions provided by the users or identified at the facility. Unless otherwise indicated, crystallization experiments were set up with 100 nl sample and 100 nl crystallization solution on the inner surface of the films within a CrystalDirect plate using a Cartesian PixSys robot (Cartesian Technologies). The reservoirs contained 45 µl crystallization solution prepared manually with a Formulator-16 robot (Fomulatrix Inc.) or with an Evoware (Tecan) liquid-handling robot. The plates were sealed on their upper side with CrystalClear film (Hampton Research) and the experiments were incubated at either 20 or 5°C in a RockImager system (Formulatrix Inc.) and regularly imaged under either under visible or UV light.

The human 8-oxoguanine DNA glycosylase (OGG1) protein sample was provided by Dr Bjørn Dalhus and Mari Ytre-Arne (University of Olso, Norway). The wild-type truncated OGG1 (12–323) was produced and purified as described previously (Bjørås *et al.*, 2002 ▸). Briefly, OGG1 containing an N-terminal hexahistidine tag was transformed in *Escherichia coli* BL21(DE3) RIL Codon Plus cells from Stratagene and overexpressed upon the addition of 1 m*M* IPTG for 18 h at 18°C. Purification steps included nickel-affinity chromatography and ion-exchange chromatography. Purified OGG1 was concentrated to 10 mg ml^−1^ using a 10 kDa cutoff centrifugal filter. The final protein buffer consisted of 20 m*M* MES–HCl pH 6.0, 50 m*M* sodium chloride, 0.5 m*M* EDTA, 10 m*M* β-mercaptoethanol. Sitting-drop crystallization experiments using a reservoir consisting of 0.1 *M* sodium citrate pH 5.5, 0.2 *M* ammonium sulfate, 24% PEG 3350 were set up at 4°C.

The transthyretin (TTR) sample was provided by Dr Trevor Forsyth and Alycia Yee (Institut Laue Langevin, Grenoble, France). It was produced and purified as described previously (Haupt *et al.*, 2014 ▸). Briefly, TTR with an N-terminal hexahistidine tag and TEV cleavage site was overexpressed in *E. coli* BL21(DE3) cells (Invitrogen). Purification was carried out using a nickel-affinity chromatography column, TEV cleavage and subsequent gel filtration. Crystals were grown using 0.1 *M* sodium citrate pH 5.0, 1.6 *M* ammonium sulfate as the crystallization buffer.

The strawberry Fra a 2-F141 protein was provided by Professor Victoriano Valpuesta, Dr Ana Casañal and Delphine Pott (University of Malaga, Spain). The protein was cloned in the pETM11 vector and was expressed as an N-terminally 6×His-tagged, TEV-cleavable fusion protein in *E. coli* BL21(DE3) cells. 2 l of *E. coli* cells were harvested after overnight induction at 20°C, lysed and loaded onto a nickel-affinity chromatography column. Following digestion with TEV protease, the N-terminal tag was removed using a second nickel-affinity chromatography step. The sample was subjected to gel filtration using a buffer consisting of 0.03 *M* Tris–HCl pH 7.5, 0.15 *M* sodium chloride, 1 m*M* β-mercapto­ethanol. Crystals appeared at a protein concentration of 38.6 mg ml^−1^ using 0.1 *M* bis-tris–HCl pH 5.5, 0.2 *M* ammonium sulfate, 25% PEG 3350 as the crystallization solution.

The Vps34 (human class III phosphoinositide 3-kinase) protein was prepared and crystallized as described by Pasquier *et al.* (2015 ▸). Briefly, Vps34 was purchased from Sprint Bioscience (Stockholm, Sweden) and concentrated to 10 mg ml^−1^ in a buffer consisting of 20 m*M* HEPES–NaOH pH 7.5, 100 m*M* sodium chloride, 1 m*M* TCEP. The ligand (2*S*)-8-[(3*R*)-3-methylmorpholin-4-yl]-1-(3-methyl-2-oxobutyl)-2-(trifluoromethyl)-3,4-dihydro-2*H*-pyrimido(1,2-*a*)pyrimidin-6-one (compound 3) was supplemented to a final concentration of 1 m*M*. After overnight incubation, crystallization drops were set up manually in CrystalDirect plates by mixing 1 µl sample and 1 µl crystallization solution consisting of 0.1 *M* Tris–HCl pH 7.5, 1.8 *M* ammonium sulfate.

Human recombinant CDK2 (cyclin-dependent kinase 2) was produced by the Protein Production Department at Sanofi. Briefly, CDK2 was overexpressed in Sf21 insect cells by infection with recombinant baculovirus. After mechanical lysis, the protein was purified by successive negative-ion, hydroxy­apatite and ATP–agarose chromatography. The CDK2 protein was conditioned in a buffer consisting of 20 m*M* HEPES–NaOH pH 7.5, 50 m*M* NaCl, 1 m*M* DTT by three successive cycles of dilution and concentration with an Amicon Ultrafree filtration device (10 000 Da cutoff). In the final cycle, the protein was concentrated to 10 mg ml^−1^ and passed through a 0.2 µm Spin-X filter. Initial CDK2 crystals were obtained with a crystallization buffer consisting of 0.02 *M* HEPES–NaOH pH 7.0, 5% glycerol, 12% PEG 3350. Crystalline material from these experiments was recovered, ground and used in microseeding experiments. The crystals used for the soaking experiments were produced either manually or with the help of a crystallization robot using the protocol described above but with the addition of 1/10 (relative to the final drop volume) of seed solution (different seed dilutions were empirically tested). For control manual soaking and mounting experiments, CDK2 crystals were manually transferred to a 4 µl drop consisting of 0.02 *M* HEPES–NaOH pH 7.0, 5% glycerol, 12% PEG 3350 and 0.2 µl ligand solution (500 m*M* in 100% DMSO). After incubation, the crystals were manually transferred to a cryosolution consisting of 0.02 *M* HEPES–NaOH pH 7.0, 20% glycerol, 12% PEG 3350 and 10 m*M* ligand solution, mounted on a data-collection pin and cryocooled in a LN2 jet.

Lysozyme from hen egg white (catalogue No. L6876), thaumatin from *Thaumatococcus daniellii* (catalogue No. T7638) and proteinase K from *Tritirachium album* (catalogue No. P6556) were purchased from Sigma–Aldrich and dissolved in water to final concentrations of 80, 40 and 20 mg ml^−1^, respectively. The crystallization solution for lysozyme was 0.1 *M* sodium acetate pH 4.6, 1.25 *M* sodium chloride. The crystallization reservoir for thaumatin consisted of 0.1 *M* HEPES–NaOH pH 7.5, 1.2 *M* sodium/potassium tartrate and that for proteinase K consisted of 0.1 *M* HEPES–NaOH pH 7.5, 1.3 *M* ammonium sulfate, 0.1 *M* sodium chloride. For control experiments, the crystals of lysozyme, thaumatin and proteinase K were manually transferred to a 2 µl drop containing the respective reservoir solution and 20% glycerol for cryoprotection. After a short incubation, they were manually mounted on a nylon loop and flash-cooled in liquid nitrogen.

### 2.2. The CrystalDirect crystal-harvesting and processing station   

The CrystalDirect robotic station (Arinax, Grenoble, France) uses a Satsuma femtosecond laser (Amplitude Systems) controlled by a laser scanner (Sunny Technology), a motorized plate stand and a robotic arm to manipulate the plates and pins. Chemical delivery experiments were performed with either a 0.5 µl precision syringe (Hamilton) mounted on a micromanipulator or one of three PipeJet (BioFluidix) nanovolume droplet dispensers integrated into the system. The system was operated as described in §[Sec sec3]3. For the CDK2 ligand-delivery experiments, the Hamilton syringe was loaded with of 0.5 µl ligand solution (500 m*M* in 100% DMSO) and aligned with the working area of the laser with the help of the micromanipulator. Drops of 50 nl ligand solution were deposited on top of the laser-generated apertures. After incubation for 3 h to overnight, the crystals were harvested and cryocooled using the automated CrystalDirect approach. Each ligand was delivered to at least two independent crystallization drops, and two or more crystals, harvested on a single or multiple pins, were analyzed by X-ray diffraction for each of the ligands.

### 2.3. Data collection and structure determination   

Diffraction data were collected on the BM14, ID14-EH4, ID29, ID23-EH1 or ID23-EH2 beamlines at the European Synchrotron Radiation Facility (ESRF; Grenoble, France). Crystallo­graphic data reduction and scaling was performed with the *XDS* software (Kabsch, 2010 ▸). Initial phases were obtained by the molecular-replacement method using *MOLREP* (Vagin & Teplyakov, 2010 ▸) and the following search models from the Protein Data Bank: PDB entries 1hcl (Schulze-Gahmen *et al.*, 1996 ▸) for CDK2, 1ko9 (Bjørås *et al.*, 2002 ▸) for OGG1, 4pvl (Haupt *et al.*, 2014 ▸) for TTR, 4c9c (Casañal *et al.*, 2013 ▸) for Fra a 2, 4uwg (Pasquier *et al.*, 2015 ▸) for Vps34, 4b0d (Cipriani *et al.*, 2012 ▸) for lysozyme, 2prk (Betzel *et al.*, 1988 ▸) for proteinase K and 4axr (Cipriani *et al.*, 2012 ▸) for thaumatin. Successive rounds of automatic refinement and manual building were carried out with *REFMAC*5 (Murshudov *et al.*, 2011 ▸) and *Coot* (Emsley *et al.*, 2010 ▸). Initial solvent models were established with *ARP*/*wARP* (Langer *et al.*, 2008 ▸) and refined manually. *MolProbity* (Chen *et al.*, 2010 ▸) was used to evaluate the general quality of the final models. Structure factors and atomic models for all of the structures discussed in this work have been deposited in the Protein Data Bank (PDB entries 5amz, 5amx, 5ebh, 5ano, 5anl, 5amw, 5an4, 5dwp, 5ank, 5anj, 5ani, 5ang, 5ane and 5and).

## 3. Results   

### 3.1. A novel approach for crystal mounting, soaking and cryocooling   

**Figure 1:**
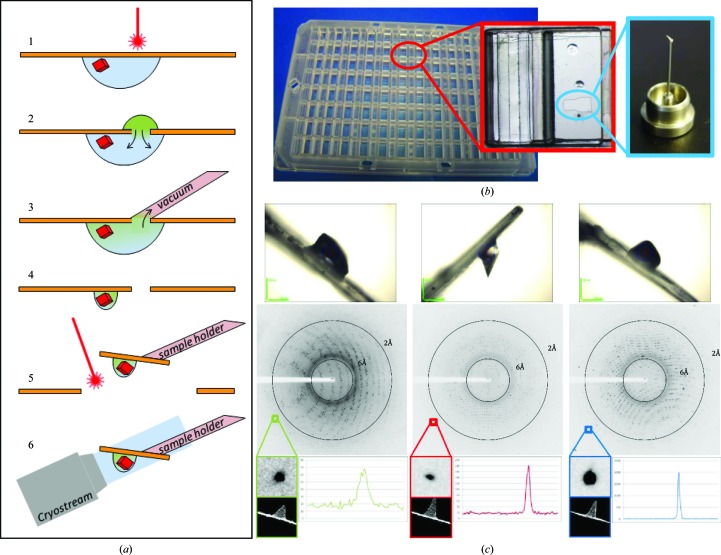
The CrystalDirect approach. (*a*) Schematic representation of the methods for automated crystal harvesting, chemical delivery and cryocooling. From top to bottom, crystals are grown on the surface of a low X-ray-background film which is directly compatible with diffraction data collection. A laser beam operating in the photoablation regime is used to produce an aperture in the film (1). Chemicals can be delivered to crystals through diffusion by applying a small amount of solution on the outside of the opening that enters into contact with the crystallization drop (2). After incubation, or immediately after producing the aperture, if no chemicals are delivered both the externally applied and the crystallization solution are gently aspirated through the aperture by applying a vacuum (3–4). The sample can then be mounted by excising the film around the crystal with the laser and gluing it to the tip of a data-collection pin (5). The crystal is then moved to a cryojet for flash-cooling (6). (*b*) The 96-well CrystalDirect microplate. One of the cells of the microplate is shown in detail (outlined in red). The reservoir containing the crystallization solution (left) and the film used as the crystallization support (right) with three crystallization drops on it can be appreciated. Crystals from one of the drops have been harvested and mounted on a pin (blue outline). (*c*) X-ray diffraction analysis of crystals of proteinase K (left), thaumatin (middle) and lysozyme (left) prepared using the automated CrystalDirect harvesting and cryocooling method. The top panels show a detail of the mounted samples as seen through the on-axis beamline camera (the blue circle indicates the size and position of the X-ray beam). Only a small amount of liquid remains between the film and the crystal after the liquid-removal step, facilitating direct cryocooling. X-ray diffraction images from these samples (middle panels) show a complete absence of ice rings and show reflections extending to high resolution with spot profiles (bottom panels) and crystallographic statistics comparable to those obtained with crystals processed with the standard manual methods (see Table 1 ▸).

The CrystalDirect approach for automated crystal harvesting and processing is illustrated in Fig. 1 ▸(*a*). Briefly, a redesigned 96-well vapour-diffusion crystallization microplate, the CrystalDirect plate (Fig. 1 ▸ *b*), is used to grow crystals in drops deposited on the surface of a thin, low X-ray-background film.

A laser beam is used to create an aperture in the film through which the crystallization solution can be removed by gentle aspiration prior to crystal mounting (steps 1 and 3 in Fig. 1 ▸ *a*). A controlled amount of glue is added to the tip of an X-ray data-collection pin (not shown in Fig. 1 ▸ *a*), which is then placed in contact with the outer face of the film in a location close to the ‘naked’ crystal (steps 4 and 5 in Fig. 1 ▸ *a*). The laser is used to excise the film around the sample, which is flash-cooled by transferring the pin to a cryocooling jet (steps 5 and 6 in Fig. 1 ▸ *a*). In a variation of this approach, chemicals can be delivered to crystals by dispensing a drop over the laser-generated aperture from the outside of the plate (step 2 in Fig. 1 ▸ *a*). In this way, the externally applied solution enters into contact with the crystallization drop and delivery of the chemical to the crystals occurs through diffusion. After incubation, both the external and internal solutions are removed by aspiration and the samples are mounted and cryocooled (steps 3–6 in Fig. 1 ▸ *a*).

### 3.2. The CrystalDirect plate and crystal-harvesting robot   

The CrystalDirect system relies on the use of a modified vapour-diffusion crystallization microplate with a 25 µm-thick cyclic olefin copolymer (COC) film as a crystallization support (Fig. 1 ▸ *b*). COC is an amorphous polymer made of carbon and hydrogen which minimizes both X-ray absorption and background scattering, is compatible with common crystallization reagents and is also transparent to both visible and UV light (Soliman *et al.*, 2011 ▸). CrystalDirect plates adopt the SBS standard and are compatible with popular crystallization robots and imaging systems.

**Figure 2:**
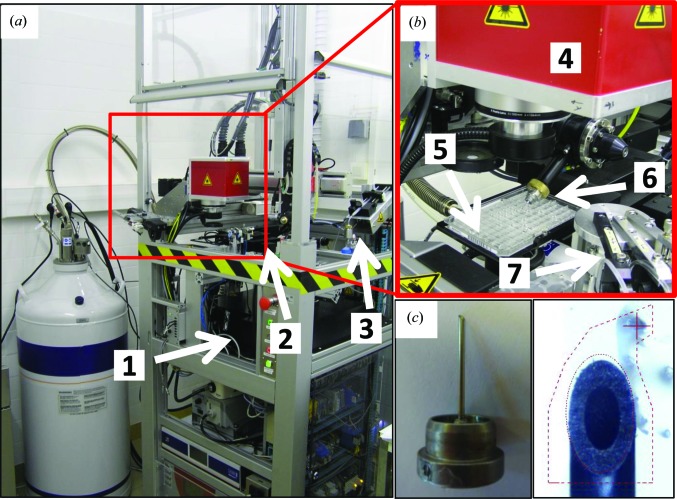
The CrystalDirect harvesting and processing robot. (*a*) General view of the crystal-harvesting and processing robot. Its dimensions are 0.8 × 0.8 × 2.3 m (length × width × height). The locations of the laser generator (1), the cryocooling jet (2) and the glue-delivery station (3) are indicated relative to the laser working area (highlighted by a red square). (*b*) Detail of the laser working area including the optical laser scanner (4), the motorized stage holding a CrystalDirect plate (5), a robotic arm handling X-ray data-collection pins (6) and a motorized stage with three PipeJet droplet dispensers (BioFluidix) (7). (*c*) Detail of the SPINE-compatible X-ray data-collection pin. In the right panel, the pin is shown right before laser excision for crystal harvesting (the picture is taken through the harvester control software). The hollow inner shaft of the pin and the selected cut shape (dotted red lines) can be appreciated.

The CrystalDirect harvesting and processing robot is shown in Fig. 2 ▸. It is composed of a 96-well microplate-handling station, a femtosecond laser source, a scanner that steers the laser beam, a robotic arm for handling pins, a glue-delivery station and a cryocooling jet (Márquez & Cipriani, 2014 ▸). The plate is positioned in a holder in an inverted orientation, *i.e.* with the crystallization film towards the upper side and the crystallization drops hanging from it (as indicated in Fig. 1 ▸ *a*). The system integrates a precision syringe (Hamilton) mounted on a micromanipulator and three PipeJet (BioFluidix) nanovolume droplet dispensers for the delivery of solutions (Fig. 2 ▸ *b*). The CrystalDirect harvester uses a SPINE-compatible X-ray data-collection pin (Cipriani *et al.*, 2006 ▸) with a bevelled tip to increase the surface of contact with the film and a hollow shaft to facilitate aspiration of the crystallization solution (Fig. 2 ▸ *c*). The harvested crystals can be stored in SC3 pucks, Uni-Pucks or any other SPINE-compatible system.

### 3.3. Automated crystal harvesting and cryocooling   

**Figure 3:**
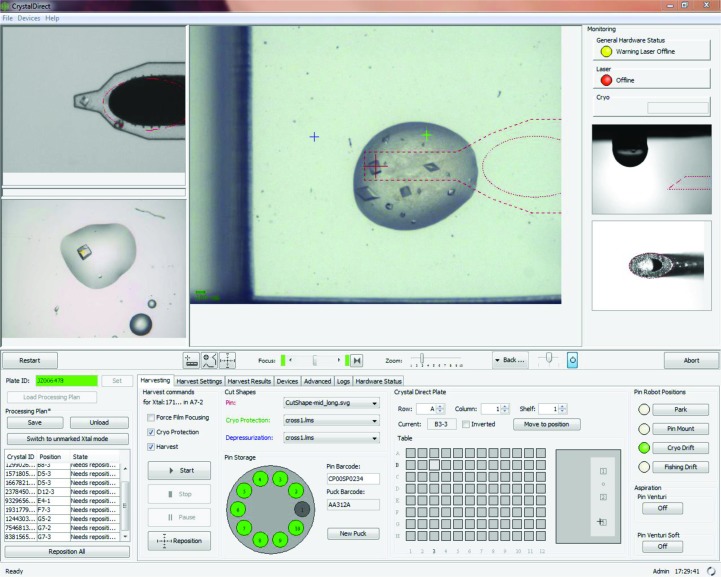
CrystalDirect robot control software. The signal from a video camera monitoring the laser working area within a microplate (centre) is used to locate the aperture (green trace) and the cutting shape (red line) and to select the final position of the tip of the pin relative to the crystal (red oval). Another camera monitors the position of the cryocooling jet (top left), allowing inspection of the samples at the end of the harvesting and cryocooling process. Images and coordinates of crystals can be stored and automatically recovered by the harvester software (bottom left). Alternatively, operators can select any position within a 96-well microplate (bottom right). The software provides full control over the harvesting and processing parameters and records the final location of the harvested samples within standard SPINE sample pucks (bottom, centre). The system also monitors a number of processes such as, for example, calibration of the pin position and delivery of a glue droplet (right middle).

The CrystalDirect plate and harvesting robot enable automation of the crystal-mounting and cryocooling process. The robot control software integrates the video signal from two cameras monitoring the laser working area and cryojet position and provides convenient interfaces to navigate through a 96-well plate and select all of the parameters required for harvesting (Fig. 3 ▸). These include the position of the pin relative to the crystal, the position and shape of the aperture for liquid aspiration and the size and shape of the film that will be excised to mount the sample. As an initial validation, crystals of proteinase K, thaumatin and lysozyme were prepared in CrystalDirect plates. These crystals grow from solutions that typically require the addition of cryoprotectants when mounted manually. The crystallographic analysis of these samples consistently showed a complete absence of ice rings, which is indicative of successful vitrification (Fig. 1 ▸ *c*), with resolution, mosaicity and overall crystallographic and refinement statistics comparable to those obtained for crystals of similar size mounted manually and subjected to a standard cryoprotection treatment with glycerol as an antifreezing agent (see Table 1 ▸ for crystallographic and refinement statistics). The automated harvesting and cryocooling sequence for a representative sample can be appreciated in Supplementary Movie S1.

**Figure 4:**
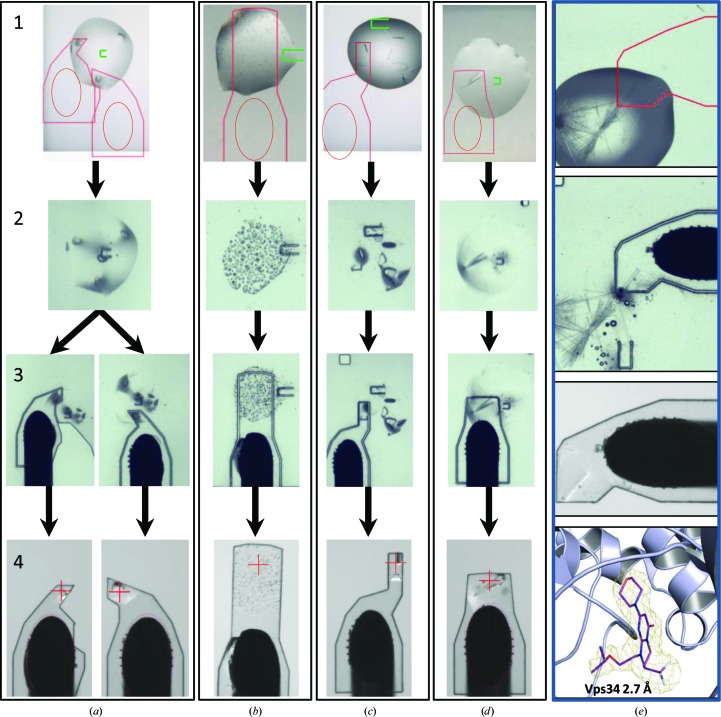
Automated crystal mounting and cryocooling. (*a*–*d*) A series of images taken along the time course of the automated harvesting process are shown. (1) The crystallization drop before harvesting; the location of the pin (round oval), the aperture (U-shapes, green) and the cutting area have been selected. (2) The aspiration aperture has been created and the crystallization solution removed by aspiration. (3) The pin has been applied to the film and the laser excision has been performed; the samples are ready for transfer to the cryojet. (4) Cryocooled samples as seen from the camera at the cryojet position. As can be appreciated, crystals tend to remain in position throughout the mounting process. This makes it possible to perform sequential harvesting operations from a single drop (*a*) or to mount multiple crystals on a single pin (*b*, *c*). (*e*) Crystal surgery: crystals of the Vps34 lipid kinase in complex with compound 3 consistently grow as tight needle-like clusters (top panel). The laser was used to cut and mount a single crystal fragment (the time course is shown from top to bottom). The full process is shown in Supplementary Movie S3. X-ray helical data collection from this sample on the ESRF ID23-2 microfocus beamline produced a high-resolution structure. The bottom panel shows a region of the electron-density OMIT map contoured at 1σ around the compound 3 binding site. Complete data-collection statistics are described in Table 2 ▸.

The CrystalDirect system is currently in operation at the High Throughput Crystallization Laboratory (HTX lab) of the EMBL Grenoble Outstation (https://embl.fr/htxlab), where it has been used to process samples provided by regular users of this facility. This has allowed us to test this approach in a number of different scenarios often found in crystallography projects and involving a variety of crystallization conditions. Figs. 4 ▸(*a*)–4 ▸(*d*) illustrate the time course of the automated mounting process and the crystallographic analysis of five of these samples, including the human 8-oxoguanine DNA glycosylase (OGG1; Bjørås *et al.*, 2002 ▸), human transthyretin (TTR; Haupt *et al.*, 2014 ▸), the strawberry Fra a 2 protein (Casañal *et al.*, 2013 ▸) and the catalytic regions of the human cell cycle-dependent protein kinase 2 (CDK2; Schulze-Gahmen *et al.*, 1996 ▸). Some of these crystals grew in conditions that require the addition of cryoprotectants when processed by standard manual methods. However, in all cases tested the automated laser mounting and direct cryocooling after liquid removal with the CrystalDirect robot produced samples with very good diffraction properties leading to high-quality structural models (see Table 2 ▸). These results indicate that the automated harvesting and cryocooling approach presented here is a generally applicable method.

This approach has also shown several advantages over manual processing. As no tools enter the crystallization drop or make contact with the crystal during the mounting process, mechanical stress to the samples is reduced, facilitating the operation with fragile crystals such as thin plates and needles. In contrast to manual approaches, crystal size and morphology do not have an impact on the mounting process. Moreover, direct crystal cryocooling contributes to streamlining diffraction analysis in many cases, as finding appropriate cryoprotectants can sometimes be challenging. In our experience, one critical parameter is the optimal removal of the crystallization solution surrounding the crystals. Ideally, only a small amount of solution should remain in the region of contact between the crystal and the film (Figs. 1 ▸ *c* and 4 ▸ *a*–4 ▸ *d*). The type and size of the aperture as well as the aspiration conditions influence this process. Appropriate parameters can easily be selected according to the drop size and crystallization condition through the CrystalDirect harvester software interface.

As can be appreciated in Fig. 4 ▸, crystals tend to remain in the same position during the harvesting process. This offers the possibility to adjust the shape of the excised film to the size and location of the sample in order to isolate single crystals as well as to operate sequentially and mount several crystals from the same drop in separate supports (Fig. 4 ▸ *a*). Alternatively, the pin position and the cutting shape can be selected so that multiple crystals are mounted in the same support along the pin axis, thereby avoiding overlaps between samples during data collection and facilitating independent measurements from multiple crystals on the same pin (Fig. 4 ▸ *c*). This was the approach used to analyse many of the CDK2–ligand complex crystals (see below). Very small crystals (microcrystals) tend to float in the bulk of the crystallization solution rather than settle on the film and some of them may flow through the aperture during aspiration. However, a larger portion tends to deposit on the surface of the film as the solution is removed (Fig. 4 ▸ *b*). This may be a convenient procedure to prepare microcrystalline samples for serial crystallography experiments. The thorough removal of the crystallization solution also contributes to reducing the background signal, which is often critical when performing X-ray diffraction measurements with microcrystals.

Femtosecond photoablation lasers, such as that used here, produce little or no collateral damage to the sample owing to heat conduction or shock waves (Gamaly *et al.*, 2002 ▸). This property can be exploited to separate individual crystals growing in dense clusters or to eliminate flawed parts of a crystal with surgical precision. A series of ablation experiments performed on thaumatin crystals confirmed that the effects of ablation do not propagate through the crystal (Supplementary Fig. S1). This approach was applied to the analysis of crystals of the catalytic subunit of the human phosphoinositide 3-kinase Vps34 in complex with a specific inhibitor (Pasquier *et al.*, 2015 ▸). This sample consistently produces a large number of thin needles growing in clusters that are difficult to separate using the manual mounting approach (see Fig. 4 ▸ *e*). We used the laser beam as a surgical tool to cut and extract a portion of a rod-shaped crystal from a cluster of densely packed needles (see Supplementary Movie S2). Helical X-ray data collection performed on this sample on the ESRF ID23-2 microfocus beamline resulted in a high-resolution structural model providing insight into Vps34–ligand interactions (see Table 2 ▸).

### 3.4. Chemical delivery through diffusion   

**Figure 5:**
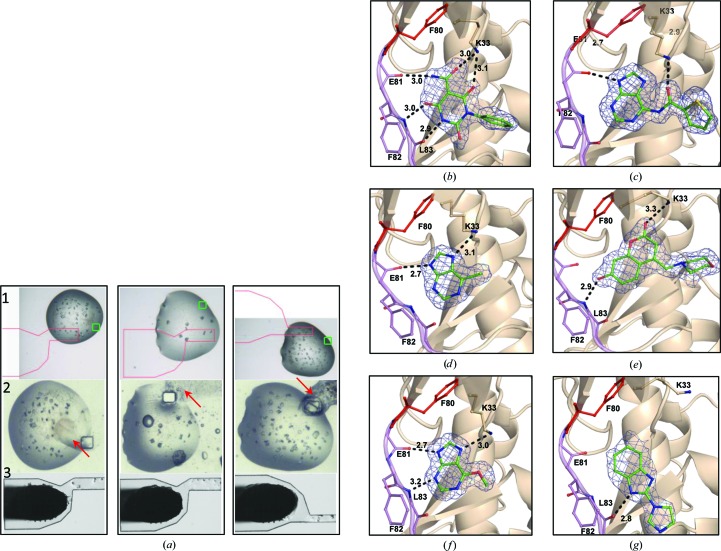
Chemical delivery through diffusion. (*a*) From top to bottom, time course of chemical delivery to three crystallization drops containing CDK2 crystals. The pictures are snapshots of the video feedback provided by the software interface of the harvesting robot (see Fig. 3 ▸). (1) The crystallization drops as seen through the robot software interface before the start; the selected locations for the aperture and the pin as well as the cutting shape are shown as in Fig. 4 ▸. In this case the aperture has a rectangular shape (green square). (2) The aperture has been generated; 50 nl ligand solution has been deposited on the outer side of the film. Occasionally, precipitation of the ligand is observed in the areas proximal to the aperture (red arrows). (3) Mounted and cryocooled samples after liquid removal, as seen from the cryojet camera. (*b*–*g*) Detail of six small molecule–CDK2 complex structures obtained from crystals prepared by this method. (*b*) 2,4,6-Trioxo-1-phenyl-hexahydropyrimidine-5-carboxamide, (*c*) *N*-(9*H*-purin-6-yl)thiophene-2-carboxamide, (*d*) 6-chloro-7*H*-purine, (*e*) 7-hydroxy-4-(morpholinomethyl)chromen-2-one, (*f*) 6-methoxy-7*H*-purine and (*g*) 2-imidazol-1-yl-1*H*-benzimidazole. The hinge region of CDK2 (Glu81–Leu83) is shown in purple, the gatekeeper residue Phe80 in red and the ligands in green; the 2*F*_o_ − *F*_c_ OMIT map contoured at 1σ is represented as a blue mesh. Hydrogen-bond interactions are denoted by black dashed lines. Complete data-collection statistics are given in Table 3 ▸.

The CrystalDirect technology offers the possibility to deliver chemicals by diffusion from a solution delivered to the top of the laser-generated aperture prior to liquid aspiration and crystal mounting (Fig. 1 ▸ *a*). A video demonstrating this principle is presented as Supplementary Movie S3. We have used this approach to analyse the binding of a collection of ten small molecules to the CDK2 protein. These molecules had been identified as potential CDK2 binders through a combination of high-throughput screening, *in silico* screening and NMR screening of a 623-fragment library. CDK2 crystals were grown in CrystalDirect plates and a 15 × 20 µm rectangular aperture was generated in selected drops containing crystals. 50 nl fragment solution was delivered on the outer side of the laser-generated aperture with a Hamilton syringe mounted on a precision stage. The solutions were allowed to diffuse for several minutes to up to 48 h. No signs of dehydration were apparent during this time, probably because the small size of the aperture relative to the volume of the crystallization cell. Moreover, vapour diffusion is expected to occur from the reservoir well, compensating for moderate losses of water vapour in the crystallization drop. Both the crystallization and the externally applied ligand solutions were removed during the aspiration step. The fragments were delivered in their original formulation (500 m*M* in 100% DMSO) in order to achieve the highest possible concentration in the experiment. In some cases, the ligand solution produced a precipitate in the area proximal to the aperture owing to the limited solubility of the molecules in the crystallization solution (Fig. 5 ▸ *a*). However, this did not seem to prevent the diffusion of the small molecules into the crystals. To avoid interference from small-molecule precipitates, we took the precaution of choosing a location for the aperture that was distant from the crystals that will be mounted. We obtained CDK2–ligand complex structures with six of the ten fragments (Figs. 5 ▸ *b*–5 ▸ *g*), while repeated experiments with the other four fragments produced only apo structures. The six CDK2–ligand complex structures show that all the molecules occupy the ATP-binding pocket and bind to the hinge region (Figs. 5 ▸ *b*–5 ▸ *g*). The resolution ranges from 2.3 to 1.6 Å (complete data-collection statistics are described in Table 3 ▸) and provide sufficient detail on the nature of the protein–ligand interactions to support the rational design of improved inhibitors based on the analysis of their binding modes. Manual soaking experiments produced equivalent results, indicating that the approach presented here produces results which are comparable to those obtained with traditional soaking methods.

## 4. Discussion   

The introduction of fast pixel-array detectors is opening new opportunities and increasing the capacity of modern synchrotron facilities for macromolecular crystallography (de Sanctis *et al.*, 2012 ▸; Hülsen *et al.*, 2006 ▸). However, the preparation of crystals for diffraction experiments still requires manual, elaborate operations that can damage the sample. The CrystalDirect system enables the automation of sample mounting and cryocooling and can contribute to closing the automation gap that currently exists between crystallization and X-ray data collection. Furthermore, by eliminating manual procedures, crystal mounting and processing becomes a more reliable and controlled operation that does not depend on the skills of the scientist. This approach can help in efficiently preparing large numbers of crystals for diffraction experiments in everyday crystallography projects and may be critical for the manipulation of samples from challenging targets with a tendency to crystallize in unfavourable morphologies.

The CrystalDirect approach provides a robust method for automated sample cryocooling without the need to screen for cryoprotectant agents, which is often a critical step in protein crystallography. The data presented here extend the previous experimental evidence (Pellegrini *et al.*, 2011 ▸) and confirm that direct sample cryocooling of ‘naked’ crystals is a generally applicable approach. By removing the external solvent, the likelihood of ice formation is reduced, as water molecules within the crystal are strongly influenced by the protein surface, which interferes with ice formation (Pellegrini *et al.*, 2011 ▸). At the same time, this helps reduce the total mass of the sample, leading to higher cooling rates. In our approach, liquid removal is carried out through gentle aspiration while the samples are still inside the crystallization well, which helps to maintain the crystals in their native environment during the process. Using the CrystalDirect system it is also possible to deliver cryoprotectant agents through the aperture prior to liquid removal. With the samples tested so far (representing more than 50 different crystallization conditions) this approach has not produced better results than the direct cryocooling method. However, it might be expected that crystals with a high solvent content might require the addition of cryoprotectant agents (Pellegrini *et al.*, 2011 ▸). In such cases, the CrystalDirect approach could provide a rapid and reproducible method to test the effects of different cryoprotectants.

A simple modification of the CrystalDirect protocol (Fig. 1 ▸ *a*) makes it possible to deliver small molecules and other chemicals to crystals by diffusion, providing an alternative to manual crystal-soaking experiments. Crystal soaking is currently applied in structure-guided drug design and to the study of small molecule–protein interactions, and typically requires the preparation and analysis of large numbers of crystals through tedious manual recovery and transfer experiments. This limits the size of the chemical libraries that are typically analysed. Another drawback associated with this approach is the low tolerance of protein crystals to the organic solvents in which small-molecule collections are typically formulated. Hence, small-molecule stock solutions often need to be diluted to maintain the final concentration of organic solvents at as low as possible (typically 5% or lower) and formulated to match the composition of the crystallization solution, which limits the final concentration of ligand in the experiment and adds handling steps. Compared with the direct transfer of the crystal to a bulk solution, delivery of chemicals through diffusion is expected to produce a more gradual change in the composition of the mother liquor surrounding the crystal and a lower level of osmotic stress, potentially limiting damage to the samples. Indeed, in the experiments presented here the ligand solutions were delivered to the diffusion aperture without dilution and in 100% DMSO. This protocol did not result in any observable degradation of the diffraction power of the crystals, while the final ligand concentrations were four times higher than those used in typical manual soaking experiments. In addition to contributing to the automation of ligand-screening experiments, the diffusion method presented here may represent a more efficient approach to deliver small molecules to crystals, helping to increase the likelihood of detecting interactions by achieving higher effective ligand concentrations. This approach could also potentially be applied to deliver other types of chemicals such as cryoprotectants or phasing agents, for example. The CrystalDirect chemical diffusion method could potentially be applied to other film-based crystallization supports, for example microfluidic chips, and can be applied without the use of the harvesting robot by piercing the film of the CrystalDirect plate with a needle or other device to create the diffusion aperture and applying a drop of solution to it.

The CrystalDirect approach could also contribute to facilitating the preparation of samples for serial crystallography experiments. This approach has been applied at X-ray free-electron lasers (Chapman *et al.*, 2011 ▸) and more recently at X-ray synchrotrons (Gati *et al.*, 2014 ▸) for the structural analysis of samples that do not yield crystals of sufficient size to be analysed by conventional methods. Initial experiments were performed with the use of sample jets (Chapman *et al.*, 2011 ▸; Weierstall *et al.*, 2014 ▸), and recent work has shown that it is also possible to perform such experiments on solid supports, limiting the amount of sample consumed (Zarrine-Afsar *et al.*, 2012 ▸; Hirata *et al.*, 2014 ▸; Cohen *et al.*, 2014 ▸). The CrystalDirect approach presented here could represent a convenient method to prepare such samples with minimal manipulations and at densities which are optimal for this type of experiment, while contributing to a better signal-to-noise ratio through the controlled removal of the solvent surrounding the crystals.

The CrystalDirect system offers an unprecedented level of control in the process of mounting crystals, opening new opportunities to exploit recent technological developments in macromolecular crystallography. As illustrated in Figs. 4 ▸ and 5 ▸, it is often possible to mount multiple crystals on a single pin while enabling optimal X-ray data collection for each of them. This capability may contribute to increasing the efficiency of modern crystallography stations; firstly by making it possible to store larger numbers of samples in current automated beamline sample storage and exchange systems and secondly by reducing the number of sample-exchange cycles, which require time and are error-prone. This can be particularly useful at beamlines equipped with fast pixel-array detectors, where currently sample exchange takes longer than data collection.

Finally, the combination of automated crystal harvesting and processing with automated multi-sample X-ray data-collection and analysis protocols (Brockhauser *et al.*, 2012 ▸; Svensson *et al.*, 2015 ▸) will enable the development of a new generation of facilities integrating crystallization, X-ray data collection and processing into highly automated workflows. Such facilities could contribute to speeding up the process of crystallographic analysis by removing difficult operations that currently require time and training. Furthermore, they could be of particular interest for projects that require the analysis of large numbers of crystals, for example those focusing on challenging targets such as multi-protein complexes or membrane proteins or those involving large-scale compound and fragment screening in the context of drug-design campaigns.

The CrystalDirect system is currently in operation at the High Throughput Crystallization Laboratory of the EMBL Grenoble Outstation, where users can send purified protein to access integrated crystallization screening and automated crystal-mounting and processing services (funded through the EC BioStructX and iNEXT projects). Moreover, both the crystallization plates and harvesting robot described here are currently available from commercial suppliers; hence, the methods presented here can also be implemented elsewhere.

## Supplementary Material

PDB reference: Fra a 2, 5amw

PDB reference: proteinase K, 5amx

PDB reference: thaumatin, 5amz

PDB reference: OGG1, 5an4

PDB reference: CDK2–ligand complexes, 5and

PDB reference: 5ane

PDB reference: 5ang

PDB reference: 5ani

PDB reference: 5anj

PDB reference: 5ank

PDB reference: Vps34, 5anl

PDB reference: CDK2, 5ano

PDB reference: TTR, 5dwp

PDB reference: lysozyme, 5ebh

Supplementary Movie S1. Automated crystal harvesting and cryo-cooling through laser photo-ablation.. DOI: 10.1107/S2059798316000954/wa5104sup5.mp4

Supplementary Movie S2. Crystal surgery.. DOI: 10.1107/S2059798316000954/wa5104sup6.mp4

Supplementary Movie S3. Chemical delivery through diffusion.. DOI: 10.1107/S2059798316000954/wa5104sup7.mp4

## Figures and Tables

**Table 1:** Crystallization conditions, data-collection and refinement statistics Values in parentheses are for the outer resolution shell.

Protein	Thaumatin	Proteinase K	Lysozyme
PDB code	5amz	5amx	5ebh
Crystallization condition	0.1 *M* HEPES, 1.2 *M* sodium/potassium tartrate pH 7.5	0.1 *M* HEPES, 1.3 *M* ammonium sulfate, 0.1 *M* NaCl pH 7.5	0.1 *M* sodium acetate, 1.25 *M* NaCl pH 4.6
Data collection			
Space group	*P*4_1_2_1_2	*P*4_3_2_1_2	*P*4_3_2_1_2
Unit-cell parameters			
*a* (Å)	57.51	67.97	78.80
*b* (Å)	57.51	67.97	78.80
*c* (Å)	150.24	102.06	37.27
α (°)	90	90	90
β (°)	90	90	90
γ (°)	90	90	90
Wavelength (Å)	0.954	0.976	0.966
Resolution (Å)	28.76–1.40 (1.48–1.40)	29.13–1.01 (1.06–1.01)	19.70–1.20 (1.22–1.20)
No. of observations			
Overall	543568	766287	312366
Unique	50733	113538	37274
Average multiplicity	10.7 (10.6)	6.7 (1.5)	8.4 (7.6)
*R*_p.i.m._	0.032 (0.354)	0.020 (0.257)	0.022 (0.298)
*R*_merge_	0.100 (0.107)	0.052 (0.304)	0.060 (0.777)
Completeness (%)	100.0 (100.0)	89.6 (38.6)	100.0 (100.0)
〈*I*/σ(*I*)〉	14.5 (2.0)	19.3 (2.0)	17.2 (2.4)
Wilson *B* factor (Å^2^)	11.1	5.1	11.3
Refinement			
Resolution (Å)	30–1.4	30–1.01	20–1.20
No. of unique reflections	48063	107762	35325
*R*_work_	0.179	0.157	0.1745
*R*_free_	0.203	0.165	0.2010
No. of non-H atoms			
Total	1809	2400	1224
Protein	1545	2036	977
Ligand/ion	—	5	—
Solvent	264	359	215
Ramachandran plot (%)			
Favoured	98.0	97.05	97.6
Allowed	2.0	2.21	1.6
Outliers	0.0	0.74	0.0
R.m.s. deviations			
Bond lengths (Å)	0.024	0.026	0.012
Bond angles (°)	2.237	1.973	1.900
Average *B* factor (Å^2^)	48.6	8.3	13.983

**Table 2:** Crystallization conditions, data-collection and refinement statistics Values in parentheses are for the outer resolution shell.

Protein	CDK2	Vps34	Fra a 2	OGG1	TTR
PDB code	5ano	5anl	5amw	5an4	5dwp
Crystallization condition	0.02 *M* HEPES, 5% glycerol, 12% PEG 3350 pH 7.0	0.1 *M* Tris, 1.8 *M* ammonium sulfate pH 7.5	0.1 *M* bis-tris, 0.2 *M* ammonium sulfate, 25% PEG 3350 pH 5.5	0.1 *M* sodium citrate, 0.2 *M* ammonium sulfate, 24% PEG 3350 pH 5.5	0.1 *M* sodium citrate, 1.6 *M* ammonium sulfate pH 5.0
Data collection					
Space group	*P*2_1_2_1_2_1_	*P*2_1_2_1_2	*C*2	*P*6_5_	*P*2_1_2_2_1
Unit-cell parameters					
*a* (Å)	53.30	88.21	105.56	106.07	41.35
*b* (Å)	71.57	145.76	41.46	106.7	62.52
*c* (Å)	72.03	61.47	92.06	47.29	85.43
α (°)	90	90	90	90	90
β (°)	90	90	116.28	90	90
γ (°)	90	90	90	90	90
Wavelength (Å)	0.978	0.976	0.976	0.979	0.976
Resolution (Å)	29.84–1.70 (1.79–1.70)	29.54–2.70 (2.85–2.70)	27.51–1.90 (2.00–1.90)	27.00–1.60 (1.69–1.60)	19.63–1.20 (1.26–1.20)
No. of observations					
Overall	203444	114617	132522	282648	317175
Unique	31059	22467	28520	40235	69742
Average multiplicity	6.6 (6.6)	5.1 (5.1)	4.6 (4.7)	7.0 (7.0)	4.5 (4.4)
*R*_p.i.m._	0.026 (0.367)	0.062 (0.340)	0.035 (0.357)	0.032 (0.231)	0.040 (0.532)
*R*_merge_	0.062 (0.873)	0.130 (0.706)	0.068 (0.699)	0.082 (0.587)	0.068 (0.691)
Completeness (%)	100.0 (100.0)	99.9 (100.0)	99.9 (100.0)	99.9 (100.0)	99.7 (99.5)
〈*I*/σ(*I*)〉	16.0 (1.6)	9.6 (2.1)	12.3 (2.2)	14.8 (3.0)	8.2 (1.3)
Wilson *B* factor (Å^2^)	23.2	36.1	28.5	12.6	10.8
Refinement					
Resolution (Å)	30–1.70	30–2.70	30–1.90	27.00–1.60	19.63–1.2
No. of unique reflections	29444	21323	27078	38279	66062
*R*_work_	0.204	0.208	0.221	0.184	0.162
*R*_free_	0.255	0.267	0.265	0.214	0.195
No. of non-H atoms					
Total	2372	4416	2682	2586	2154
Protein	2229	4304	2498	2419	1792
Ligand/ion	—	27	—	10	—
Solvent	143	85	182	157	244
Ramachandran plot (%)					
Favoured	97.4	96.83	97.8	96.13	99.6
Allowed	2.2	2.98	2.19	3.55	0.4
Disallowed	0.4	0.19	0.0	0.32	0.0
R.m.s. deviations					
Bond lengths (Å)	0.019	0.012	0.017	0.022	0.012
Bond angles (°)	1.914	1.547	1.838	1.952	1.732
Average *B* factor (Å^2^)	38.1	54.4	35.0	18.6	17.9

**Table 3:** Data-collection and refinement statistics for CDK2–ligand complexes Values in parentheses are for the outer resolution shell.

Compound shown in	Fig. 5 ▸(*b*)	Fig. 5 ▸(*c*)	Fig. 5 ▸(*d*)	Fig. 5 ▸(*e*)	Fig. 5 ▸(*f*)	Fig. 5 ▸(*g*)
PDB entry	5ank	5anj	5ani	5ang	5ane	5and
Data collection						
Space group	*P*2_1_2_1_2_1_	*P*2_1_2_1_2_1_	*P*2_1_2_1_2_1_	*P*2_1_2_1_2_1_	*P*2_1_2_1_2_1_	*P*2_1_2_1_2_1_
Unit-cell parameters						
*a* (Å)	69.05	53.67	53.17	53.71	52.63	53.61
*b* (Å)	51.72	71.21	71.06	71.82	71.32	70.56
*c* (Å)	71.26	72.23	72.27	72.35	72.04	72.44
α (°)	90	90	90	90	90	90
β (°)	90	90	90	90	90	90
γ (°)	90	90	90	90	90	90
Wavelength (Å)	0.972	1.033	1.033	1.033	0.966	1.033
Resolution (Å)	29.34–1.90 (2.0–1.90)	29.96–1.6(1.69–1.6)	29.89–1.9 (2.0–1.9)	29.85–1.9 (2.0–1.9)	29.73–1.7 (1.79–1.7)	29.47–2.3 (2.42–2.3)
No. of observations						
Overall	133741	243414	119924	147308	223387	82403
Unique	20768	37295	22112	22707	30471	12740
Average redundancy	6.4 (6.2)	6.5 (6.6)	5.4 (2.2)	6.5 (6.3)	7.3 (7.4)	6.5 (6.5)
*R*_p.i.m._	0.028 (0.306)	0.031 (0.328)	0.038 (0.380)	0.031 (0.205)	0.046 (0.257)	0.058 (0.412)
*R*_merge_	0.066 (0.706)	0.074 (0.781)	0.082 (0.787)	0.073 (0.475)	0.119 (0.662)	0.135 (0.972)
Completeness (%)	99.9 (99.8)	100 (100)	99.6 (99.7)	99.9 (99.7)	99.8 (99.6)	99.9 (100)
〈*I*/σ(*I*)〉	11.5 (2.4)	14.1 (2.3)	10.9 (2.0)	15.0 (3.6)	10.9 (2.3)	7.9 (2.0)
CC_1/2_	0.999 (0.828)	0.999 (0.739)	0.998 (0.692)	0.998 (0.910)	0.998 (0.797)	0.995 (0.586)
Wilson *B* factor (Å^2^)	34.4	16.1	31.9	23.5	13.2	40.3
Refinement						
Resolution (Å)	30–1.90	29.67–1.6	30–1.9	30–1.9	30–1.7	30–2.3
No. of unique reflections	19657	35378	20946	21503	28887	12067
*R*_work_	0.194	0.196	0.187	0.196	0.204	0.202
*R*_free_	0.236	0.229	0.235	0.235	0.251	0.238
No. of non-H atoms						
Total	2337	2499	2352	2256	2550	2291
Protein	2204	2336	2254	2155	2366	2194
Ligand	18	17	10	19	11	14
Solvent	115	146	88	82	173	83
Ramachandran plot						
Favoured	97.7	98.6	97.4	96.9	97.9	96.6
Allowed	1.9	1.4	2.6	2.7	2.1	3.0
Outliers	0.4	0.0	0.0	0.4	0.0	0.4
R.m.s. deviations						
Bond lengths (Å)	0.016	0.024	0.017	0.019	0.019	0.013
Bond angles (°)	1.826	2.188	1.792	2.066	1.962	1.605
Average *B* factor (Å ^2^)	48.6	25.2	41.1	32.9	22.9	52.3
